# Use of optimal fluoroscopic angulation to facilitate effective pulsed field ablation in a patient with atrial fibrillation

**DOI:** 10.1002/joa3.70005

**Published:** 2025-01-23

**Authors:** Shintaro Yamagami, Shumpei Mori, Tomohiro Sato, Hirokazu Kondo, Toshihiro Tamura

**Affiliations:** ^1^ Department Of Cardiology Tenri Hospital Tenri Japan; ^2^ UCLA Cardiac Arrhythmia Center, UCLA Health System David Geffen School Of Medicine At Ucla Los Angeles California USA

**Keywords:** atrial fibrillation, catheter ablation, fluoroscopic angulation, pulmonary vein isolation, pulsed field ablation

## Abstract

The circular‐shaped PulseSelect™ PFA catheter has demonstrated comparable efficacy to traditional thermal catheter ablation in achieving pulmonary vein isolation (PVI), while preventing thermally mediated complications. However, this catheter does not have any objective parameters to confirm real‐time tissue‐catheter contact. We report a case in which PVI was achieved through PFA using optimal biplane fluoroscopic angulations which were more useful for accurately assessing and adjusting the position and rotation of the circular catheter electrodes than the conventional fluoroscopic angulations.

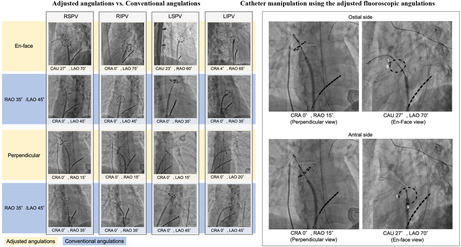

Pulsed field ablation (PFA) is a novel modality used for treating cardiac arrhythmias. Unlike thermal‐based catheter ablation systems such as radiofrequency and cryoablation, PFA nonthermally creates lesions in cardiac tissue and can achieve pulmonary vein isolation (PVI) without any collateral damage.^1^ One such device, the circular‐shaped PulseSelect™ (Medtronic, Inc., Minneapolis, MN, USA) PFA catheter, has demonstrated comparable efficacy to traditional thermal catheter ablation in achieving PVI, while preventing thermally mediated complications.^2^ However, this catheter does not have any objective parameters to confirm real‐time tissue‐catheter contact. Therefore, fluoroscopic observation is required to ensure optimal contact. Herein, we report a case in which PVI was achieved through PFA using optimal biplane fluoroscopic angulations that rendered en face and perpendicular views of each pulmonary vein (PV) ostium. Compared with the conventional fluoroscopic angulation, the customized angulations used in this case were more useful for accurately assessing and adjusting the position and rotation of the circular catheter electrodes.

A 51‐year‐old woman complaining of palpitations was referred to our department for catheter ablation due to drug‐refractory paroxysmal atrial fibrillation (AF). Routine preprocedural cardiac computed tomography (CT) was performed to evaluate the coronary artery disease status and cardiac structural anatomy, including the alignment of each PV.

The ablation procedure was performed under general anesthesia, and the airway was secured using endotracheal intubation. After transseptal puncture under intracardiac echocardiographic (ICE) guidance, the PulseSelect™ catheter was inserted into the left atrium. A biplane fluoroscopy system (Shimadzu, Kyoto, Japan) was used to isolate each PV and obtain en face and perpendicular views of each PV ostium. To achieve this, the fluoroscopic image angulations were set as closely as possible to the reference values obtained from the preprocedural cardiac CT datasets (Figure 1). Subsequently, the PulseSelect™ circular array was positioned at each PV ostium and antrum. In the perpendicular view, the catheter was pulled back from the ostial to the antral side of each PV. The antral side was identified by widening of the distance between the No. 1 and No. 9 electrodes in the en face view (Figure 2). A minimum of four applications was then delivered to each location. During each of the four applications, the catheter was repositioned in the superior/inferior/left/right directions while pointing the No. 5 electrode towards the respective direction in the en face view (Figure 2) to achieve full circumferential isolation of the PV. Complete PVI of all PVs was confirmed by entrance block testing using an Advisor™ HD Grid mapping catheter (Abbott Laboratories, Abbott Park, IL, USA). After complete PVI, no further procedures were performed. The fluoroscopy dose, total fluoroscopy time and total procedure time were 280 mGy, 20 min and 115 min, respectively, and no procedure‐related complications occurred. Furthermore, no AF recurrence was observed during the 3‐month follow‐up.

**FIGURE 1:**
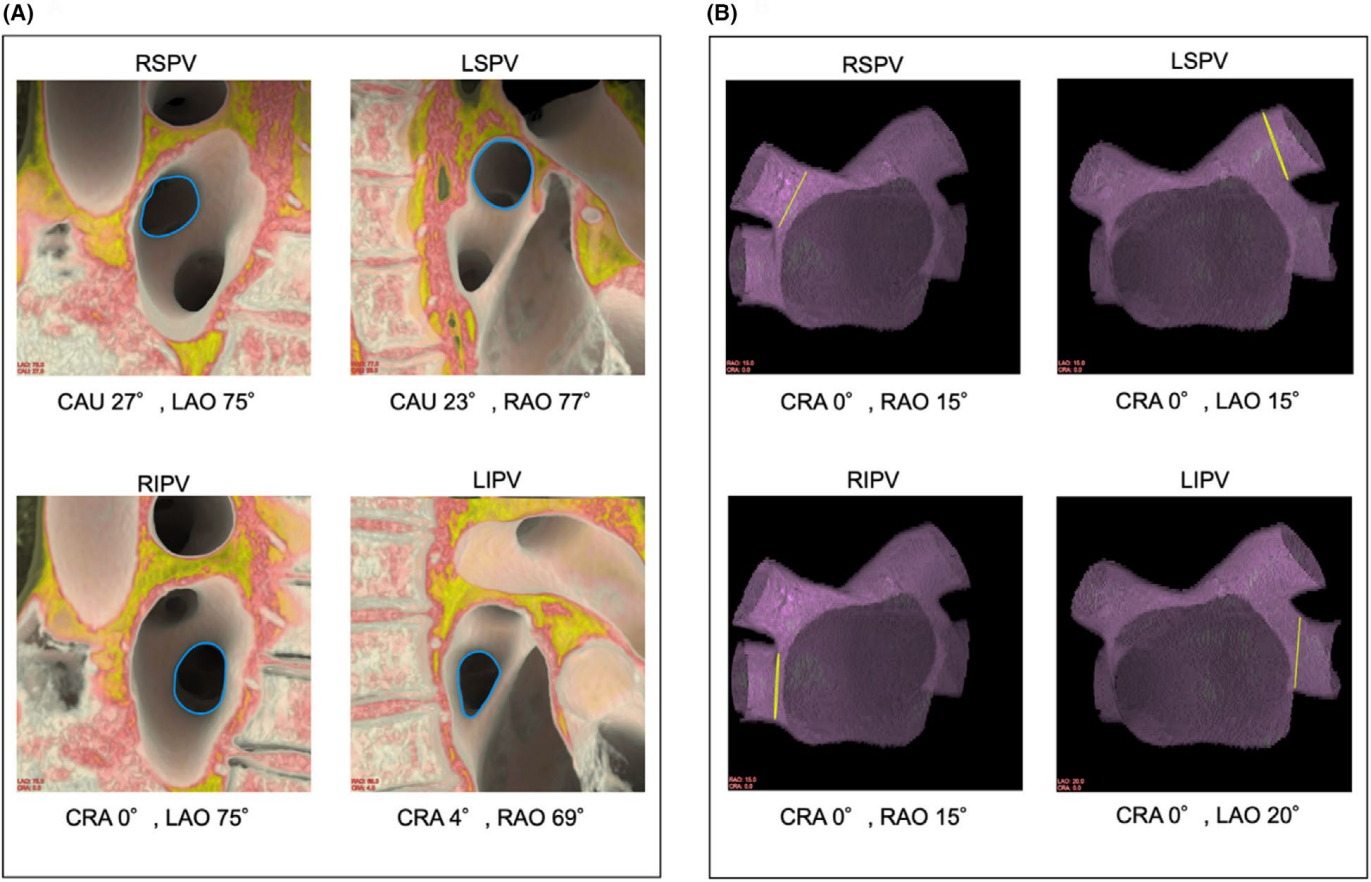
Simulated fluoroscopic angulations of the en face and perpendicular views of each PV ostium. The ostium of each PV (blue and yellow lines) is shown in the en face view (A) and perpendicular view (B), simulated by cardiac computed tomography. CAU, caudal; CRA, cranial; LAO, left anterior oblique; LIPV, left inferior pulmonary vein; LSPV, left superior pulmonary vein; PV, pulmonary vein; RAO, right anterior oblique; RIPV, right inferior pulmonary vein; RSPV, right superior pulmonary vein.

**FIGURE 2:**
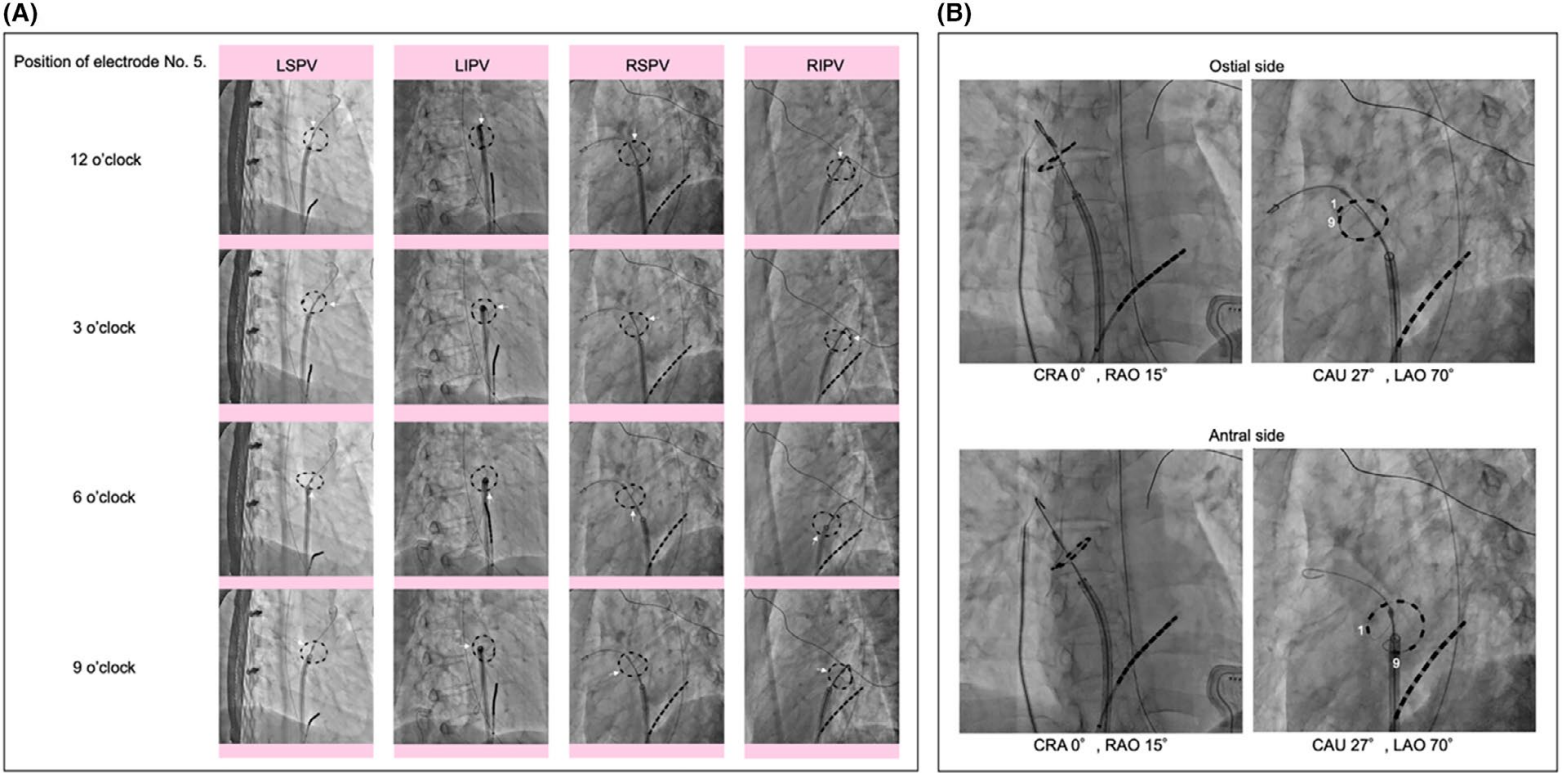
Catheter manipulation using the optimal fluoroscopic image angulations. (A) The en face view of each PV enables the intuitive and straightforward adjustment of the No. 5 electrode positions (white arrows) at 12, 3, 6, and 9 o'clock to ensure optimal contact of the catheter on the wall in each direction. (B) The perpendicular view (left panels) enables the adjustment of the catheter's position on the ostial or antral sides of the PV. When the circular‐shaped catheter is pulled back toward the antral side, enlargement of the circle (widening of the distance between the No. 1 and No. 9 electrodes) is readily confirmed by the en face view (right panels). CAU, caudal; CRA, cranial; LAO, left anterior oblique; LIPV, left inferior pulmonary vein; LSPV, left superior pulmonary vein; PV, pulmonary vein; RAO, right anterior oblique; RIPV, right inferior pulmonary vein; RSPV, right superior pulmonary vein.

Although thermal modes of ablation have been reasonably performed for years, they are limited by the potential of collateral tissue damage.^3^ In contrast, PFA nonthermally creates lesions in cardiac tissues without collateral tissue damage.^1^ Previously, the PULSED AF trial showed the efficacy of the PulseSelect™—a circular‐shaped PFA catheter—in treating AF, while not inducing any thermally mediated complications.^2^ However, this catheter does not have any objective parameters such as contact force measurements to confirm tissue‐catheter contact. Considering the linear relationship between lesion depth and electrode‐tissue proximity,^4^ the lack of sufficient tissue‐catheter contact during PFA with the PulseSelect™ can lead to ineffective lesion formation. Therefore, when using the PulseSelect™, additional steps are necessary to ensure tissue‐catheter contact.

The positioning of the circular array of the PFA catheter is generally guided by ICE, mapping systems, or fluoroscopy. ICE is especially useful for directly assessing the tissue‐catheter contact of the array visualized on the imaging plane. However, since the visualization of an en face view in each PV is technically difficult when using the PulseSelect™, the assessment of contact along the circular arrays remains challenging. Additionally, it is challenging to assess whether the catheter is on the ostial or antral side of the PV when using ICE. Mapping systems are also useful for recognizing the location and alignment of the catheters. However, skeletal muscle twitching and/or dry cough, which are frequently caused by PFA, could result in the body movement of patients and anatomical model shifting in the mapping systems. Therefore, in the present case, we optimized the fluoroscopic angulations to obtain an en face and perpendicular view of each PV by conducting a preoperative simulation using cardiac CT.

When using fluoroscopic guidance, it is a general principle to use biplane information with en face and perpendicular views of the target structure to enhance effective and safe three‐dimensional manipulation. Foreshortened views have the potential risk of misguiding procedures; this is a problem that is encountered when applying conventional right and left anterior oblique angulations for PVI, as they cannot obtain en face or perpendicular views of the PV ostia. In the present case, the intricate manipulation of the catheter became intuitive and straightforward under optimized fluoroscopic angulation (Figure 3). In fact, the en face view obtained by the adjusted fluoroscopic image angulations provided a more circular view of the PFA catheter than the conventional fluoroscopic image angulations (Figure 3). This en face view facilitated a four‐directional adjustment on the plane (Figure 2). Furthermore, the perpendicular view was useful in adjusting the position of the catheter on the ostial or antral side of the PV (Figure 2). The 20° forward‐tilted array of the PFA catheter also allows the catheter to straighten when its tip is attached to the PV antrum. This characteristic can serve as an additional indicator for confirming the catheter's attachment to the PV antrum and this could be assessed more accurately with perpendicular view. Thus, adjusting the fluoroscopic angulation for PVI according to preprocedural cardiac CT results might make fluoroscopy the easiest and most reliable tool to adjust/confirm the catheter location and alignment. This method has one notable concern—a more tilted angulation might increase the exposure of both patients and operators to radiation.^5^ Therefore, when using preprocedural simulation to predetermine the angulation, the emission of radiation should be limited to the greatest extent possible.

**FIGURE 3:**
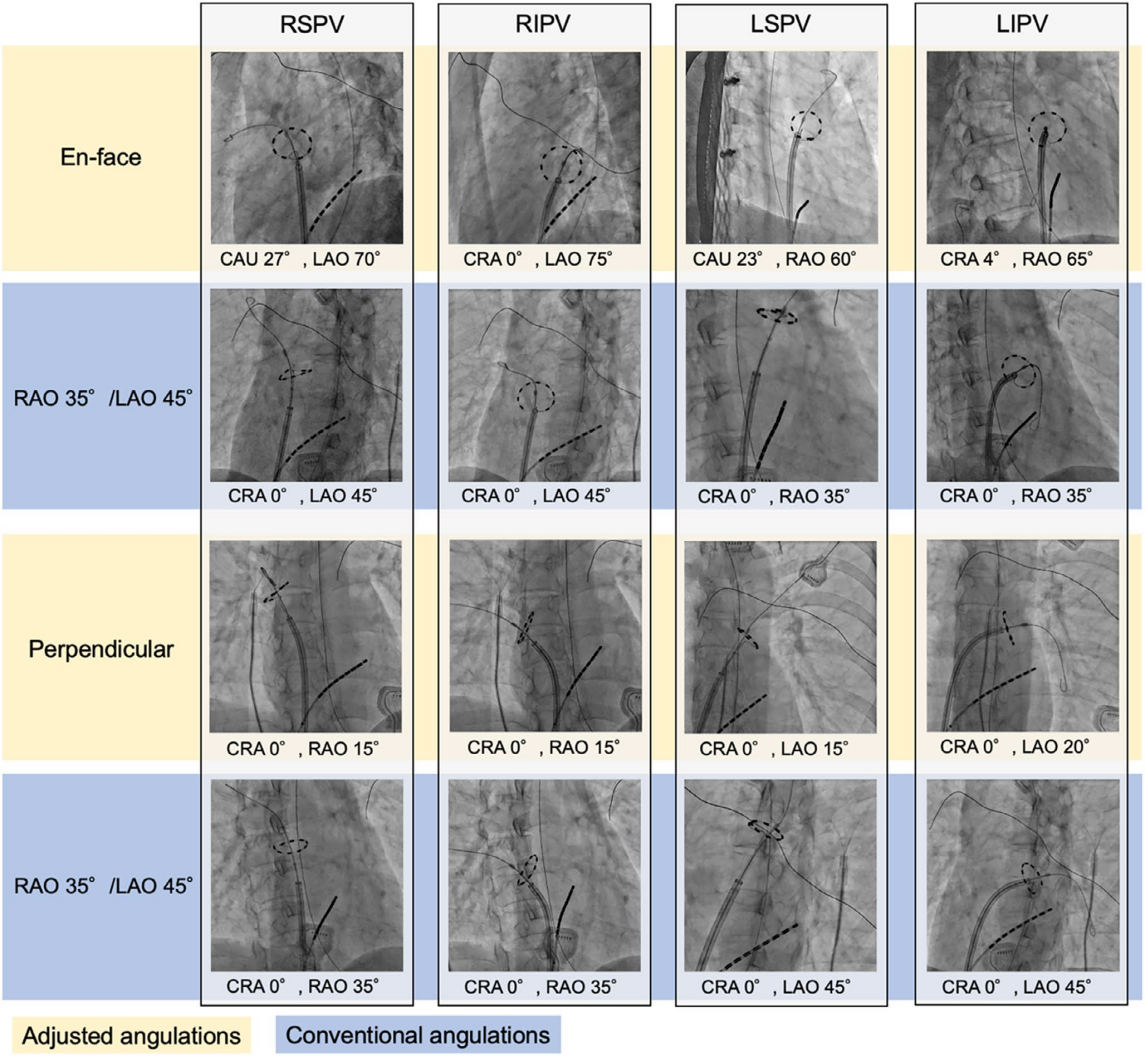
Comparison between the adjusted and conventional angulations. Compared with the conventional angulations, which generally show foreshortened images of the circular pulsed field ablation catheter, the en face and perpendicular views can provide more circular and tangential images of the catheter, respectively. This helps in the fine adjustment of the catheter's position to ensure target region selection with optimal contact. CAU, caudal; CRA, cranial; LAO, left anterior oblique; LIPV, left inferior pulmonary vein; LSPV, left superior pulmonary vein; RAO, right anterior oblique; RIPV, right inferior pulmonary vein; RSPV, right superior pulmonary vein.

Herein, we present a case of PVI performed using a PulseSelect™ PFA catheter under optimal fluoroscopic image angulation. We propose that the use of optimal fluoroscopic image angulations for each PV system could be more accurate than using conventional fluoroscopic image angulations when confirming the position and rotation of the catheter. However, further investigations are required to examine the efficacy and feasibility of this approach.

## FUNDING INFORMATION

The authors have nothing to report.

## CONFLICT OF INTEREST STATEMENT

All authors declare no conflicts of interest associated with this article.

## ETHICS STATEMENT

We have read and understood your journal's policies, and we believe that neither the manuscript nor the study violates any of these.

## Data Availability

The datasets used and/or analyzed during the current study are available from the corresponding author on reasonable request.
